# Molecular docking analysis of PPARγ antagonists for obesity associated diabetes management

**DOI:** 10.6026/97320630019633

**Published:** 2023-05-31

**Authors:** Aftab Ahmad, Anwar A. Alghamdi

**Affiliations:** 1 Health Information Technology Department, The Applied College, King Abdulaziz University, Jeddah, Saudi Arabia; 2 Pharmacovigilance and Medication Safety Unit, Center of Research Excellence for Drug Research and Pharmaceutical Industries, King Abdulaziz University, Jeddah, Saudi Arabia

**Keywords:** Obesity, PPARγ, bioactive compounds, ADMET

## Abstract

Obesity is a major metabolic disorder in developed countries, with an increasing number of people affected globally. PPARγ is primarily expressed in adipose tissue with a lesser extent in other tissues. PPARγ is an important mediator in several metabolic processes such as insulin sensitivity and adipogenesis. Because of its critical role in these processes, PPARγ is regarded as a critical target for therapeutic intervention in obesity treatment. A library of 2,320 bioactive compounds was screened insilico to identify compounds that strongly interact with the PPARγ protein. The compounds Z1982689600, Z2235802137, Z2235801970, and Z2037275165, demonstrated notable binding affinity values towards the PPARγ protein with values of -12.1, -11.7, -11.4, and -11.4 kcal/mol, respectively, which were higher than the binding affinity value observed for the control compound (-10.5 kcal/mol). These compounds bind tightly to PPARγ and have several amino acid residue interactions in common with the control compound. In addition, these compounds meet the ADMET criteria. These compounds could aid in the development of PPARγ antagonists for the management of obesity associated diabetes. However, additional research is needed to optimize their efficacy in wet laboratory conditions.

## Background:

Obesity represents a major metabolic disorder in developed nations, with a continuously growing number of individuals being affected globally. Assuming current secular trends continue, projections show that by 2030, approximately 38% of the global adult population will be classified as overweight, with an additional 20% meeting the criteria for obesity [1^1^]. Based on previous secular trends, the United States is expected to have alarming rates of overweight and obesity by 2030, with estimates indicating that more than 85% of the adult population will be affected by these conditions [2]. Contributing factors to weight gain include sedentary behaviors, unhealthy lifestyle choices, high-fat diets, genetic predisposition, and medical conditions. Moreover, obesity is also linked to other medical conditions such as dyslipidemia, diabetes, heart disease, and metabolic syndrome [3]. The economic and psychosocial burdens associated with obesity, either alone or when combined with comorbidities and related complications, are remarkably significant. Therefore, there is a pressing need for pharmacological interventions that can effectively counteract obesity and its associated metabolic complications.

Peroxisome proliferator-activated receptors (PPARs) are a subset of transcription factors that belong to the nuclear receptor superfamily. These receptors have been recognized as a critical regulator of various cellular processes involved in the pathogenesis of diabetes, obesity, and related cardiovascular disorders [4,5]. The PPAR gene family comprises three genes that encode four unique proteins: PPARα, PPARδ, PPARγ1, and PPARγ2 [6]. PPARγ is primarily expressed in adipose tissue with a lesser extent in other tissues. It serves as a critical mediator in numerous metabolic processes, including adipocyte differentiation and insulin sensitivity [7]. PPARγ is recognized as a crucial therapeutic target in the management of obesity. Experimental outcomes have demonstrated that PPARγ antagonists may have a potential role in the management of obesity and diabetes, as they have been shown to induce reductions in body weight and improvements in insulin sensitivity [8-9,10]. Therefore, it is of interest to identify novel bioactive compounds that can potentially be used as PPARγ antagonists to combat obesity associated diabetes.

## Methods:

## 3D structure retrieval and preparation of PPARγ:

The crystal structure of PPARγ [PDB ID: 4R2U] was accessed from the protein data bank [11]. In order to conduct the study, SR1664 (co-crystal ligand of 4R2U) was chosen as a positive control. The protein was then cleaned by removing both SR1664 and any water molecules present. Once the cleaning process was complete, the protein was saved in .pdb format after undergoing minimization.

## Retrieving of Bioactive compounds Library:

From the Enamine database, a bioactive library in the sdf file format containing 2320 different compounds was obtained. These compounds went through the minimization process and were put through the UFF forcefield so that they would be able to undergo docking analysis. These compounds were then converted to .pdbqt format using PyRx software [12].

## Structure based Virtual screening:

AutoDock Vina in the PyRx software was used to perform virtual screening (VS) of the prepared library (compounds). AutoDock Vina is molecular docking software that uses well-organized gradient-based optimization, and scoring functions to achieve high efficiency. It has demonstrated superior speed and accuracy, with a reported improvement of 78% over its predecessor, AutoDock 4.0 [13]. The analysis results were obtained by ranking the various inhibitor-protein complexes according to their predicted binding affinity.

## Pharmacokinetics and toxicity estimation:

Datawarrior tools had been employed to conduct an analysis of all of the compounds that were screened to carry out the preliminary assessment of physicochemical, pharmacokinetic, and drug-like properties [14].

## Result and Discussion:

**Figure 1:**
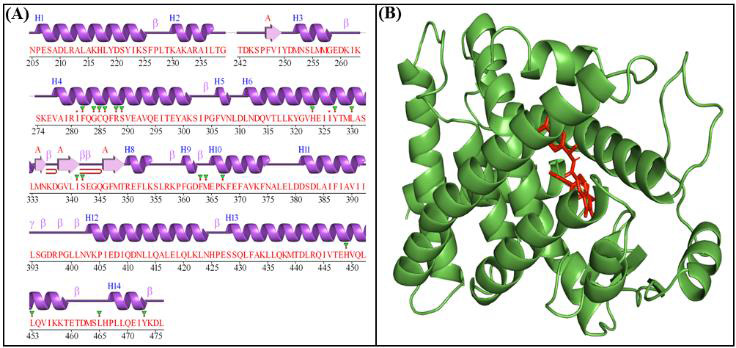
2D (A) and 3D (B) structure of PPARγ.

PPARγ plays a critical role in maintaining glucose homeostasis by promoting adipocyte differentiation, enhancing insulin sensitivity, and facilitating glucose utilization in various tissues [15]. The heterodimer 3D structure of PPARγ in complex with the SR1664 inhibitor was chosen for this study (Figure 1). The inbound ligand SR1664 inhibitor was used to calculate the grid coordinates for the X, Y, and Z-axis, which were found to be 14.35, 14.62, and 44.4, respectively.

The Enamine database was accessed in this study, yielding a collection of 2,320 different compounds. This library includes bioactive compounds that can be used in a wide range of therapeutic areas and target classes, making it one of the most comprehensive collections of its kind. These bioactive compounds include a wide range of pharmaceuticals such as central nervous system drugs, cancer drugs, and molecular glues.

**Figure 2:**
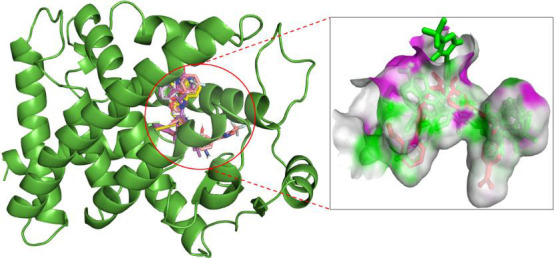
Interaction of top four hits and control compound in the active pocket of PPARγ.

After preparing the target protein structures and compound library, VS of these compounds targeting the active site residues of PPARγ was performed to identify more potent inhibitors. VS identified 35 potential leads that had higher binding energies compared to the positive control (Table 1). Based on an in-depth investigation and visualization of the docked complexes' interactions, four compounds were presented that demonstrated greater efficacy in binding by interacting with crucial PPARγ residues (Figure 2).

**Figure 3:**
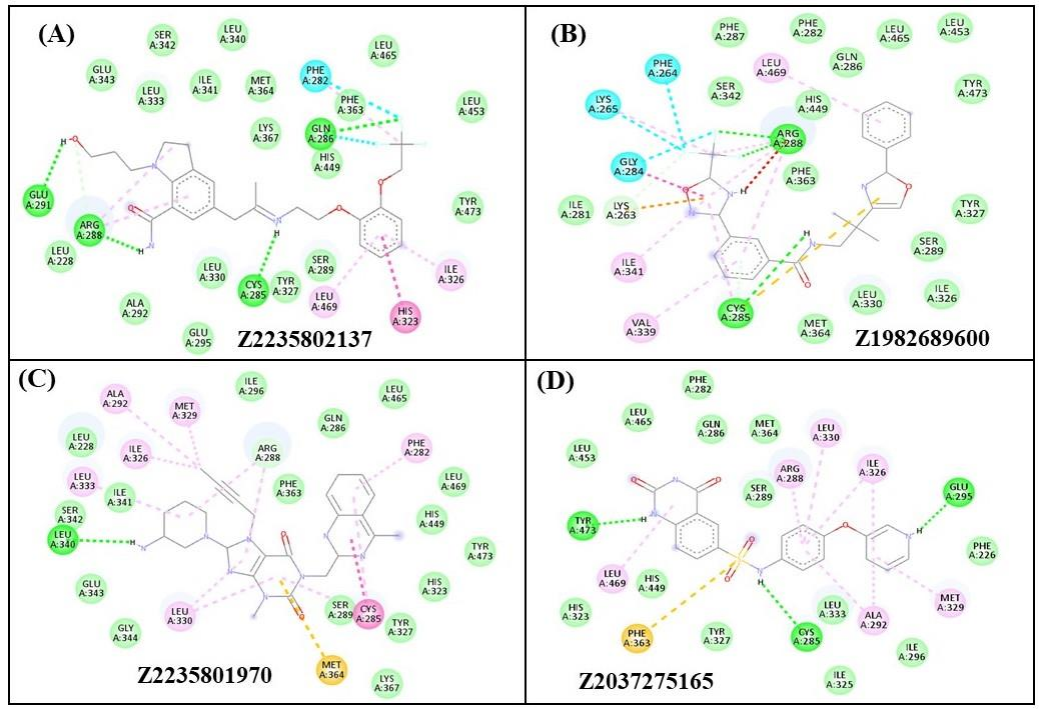
Interacting residues of Z2235802137, Z1982689600, Z2235801970, and Z2037275165 with PPARγ.

The in-depth visualization inspection of docked complexes revealed that Z1982689600, Z2235802137, Z2235801970, and Z2037275165 were found to interact in the same catalytic pocket as the positive control (Figure 2). Z2235802137 interacted with Glu291, Glu343, Ser342, Leu333, Ile341, Leu340, Met364, Lys367, Gln286, Phe282, Phe363, His449, Leu465, Leu453, Tyr473, Ile326, His323, Leu469, Ser289, Tyr327, Cys285, Leu330, Glu295, Ala292, Arg288, and Leu228 residues of PPARγ. Glu291, Arg288, Gln286, and Cys285 residues H-bonded with Z2235802137 (Figure 3A). Z1982689600 interacted with Val339, Ile341, Lys263, Ile281, Gly284, Lys265, Phe264, Phe287, Ser342, Leu469, Phe282, Gln286, His449, Arg288, Phe363, Leu465, Leu453, Tyr473, Tyr327, Ser289, Ile326, Leu330, Met364, and Cys285 residues of PPARγ. Arg288, and Cys285 residues H-bonded with Z1982689600 (Figure 3B). Z2235801970 bind with Leu330, Gly344, Glu343, Leu340, Ser342, Ile341, Leu333, Ile326, Leu228, Ala292, Met329, Ile296, Arg288, Phe363, Gln286, Leu465, Phe282, Leu469, His449, Tyr473, His323, Tyr327, Cys285, Ser289, and Met364 residues of PPARγ (Figure 3C). Further, Z2037275165 interacted with Phe363, His449, Leu469, His323, Tyr473, Leu453, Leu465, Phe282, Gln286, Met364, Arg288, Ser289, Leu330, Ile326, Glu295, Phe226, Met329, Ile296, Ala292, Leu333, Ile325, Cys285, and Tyr327 residues of PPARγ. Tyr473, Glu295, and Cys285 H-bonded with Z2037275165 (Figure 3D).

**Figure 4:**
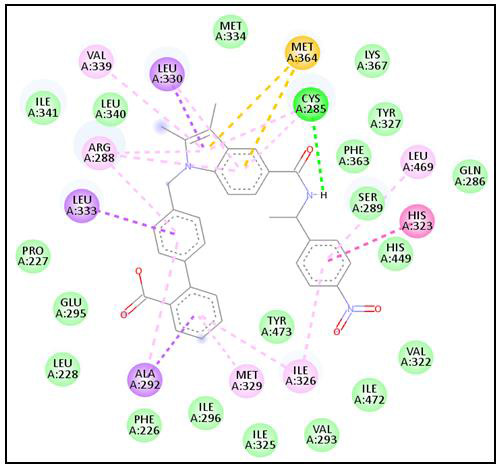
Interacting residues of SR1664 with PPARγ.

SR1664 is a PPARγ antagonist [11], and has been used as control compound in this study. SR1664 was observed to interact with Met364, Cys285, Lys367, Tyr327, Phe363, Leu469, Ser289, Gln286, His323, His449, Val322, Ile472, Val293, Ile326, Tyr473, Met329, Ile325, Ile296, Phe226, Ala292, Leu228, Glu295, Pro227, Leu333, Arg288, Ile341, Leu340, Val339, Leu330, and Met334 residues of PPARγ (Figure 4). Interestingly, Met364, Cys285, Tyr327, Phe363, Leu469, Ser289, Gln286, His449, Ile326, Tyr473, Arg288, and Leu330 were the common binding residues with the Z1982689600, Z2235802137, Z2235801970, and Z2037275165 as well as the control (SR1664) (Figure 3A-D & Figure 4). In addition, Cys285 was common H-bonded residue with Z1982689600, Z2235802137, and Z2037275165 as well as SR1664. Altogether, the results suggest that Z1982689600, Z2235802137, Z2235801970, and Z2037275165 engage in interactions with the same binding pocket on the PPARγ as the control compound SR1664.

The docking study utilized binding affinity as a metric to measure the strength of the interaction between the inhibitor-protein complexes, whereby a higher (more negative) value indicates a tighter interaction [16]. The results exhibit that Z1982689600, Z2235802137, Z2235801970, and Z2037275165 had notably higher binding affinity values relative to the control SR1664 (Table 1), pointing that these compounds have a strong affinity for binding to the PPARγ protein. The physicochemical and drug likeness properties of four selected hits were investigated using the insilico tools. The values of various physicochemical parameters, such as molecular weight, ClogP, logS, H-bond donor, H-bond acceptor, TPSA, number of rotatable bonds, and polar surface area, as well as their respective predictions, are presented in Table 2. These compounds were also found to have no serious toxicity, which involves mutagenic, tumorigenic, irritant, and reproductive effects.

## Conclusion:

In this study, a high-throughput virtual screen of a library of bioactive compounds was performed to identify compounds that interact with the PPAR protein. The compounds Z1982689600, Z2235802137, Z2235801970, and Z2037275165 were found to meet ADMET criteria and interact with key PPARγ residues, and have the potential to be useful in the development of PPARγ inhibitors. However, additional experimental testing is required to optimize their efficacy in wet laboratory conditions.

## Figures and Tables

**Table 1:** Binding affinity of top 35 compounds.

**S. No**.	**Ligand**	**Binding affinity (kcal/mol)**
1.	Z1982689600	-12.1
2.	Z2235802137	-11.7
3.	Z2235801970	-11.4
4.	Z2037275165	-11.4
5.	Z1501480424	-11.3
6.	Z2235801830	-11.3
7.	Z30442042	-11.3
8.	Z3068918473	-11.3
9.	Z1501480428	-11.2
10.	Z1521553597	-11.2
11.	Z2227698469	-11.2
12.	Z31786514	-11
13.	Z3599462428	-10.9
14.	Z195110238	-10.8
15.	Z2568726097	-10.8
16.	Z277540138	-10.8
17.	Z62954982	-10.8
18.	Z1302446275	-10.7
19.	Z1778753500	-10.7
20.	Z241910386	-10.7
21.	Z56808903	-10.7
22.	Z17617106	-10.6
23.	Z1880962221	-10.6
24.	Z27755997	-10.6
25.	Z1494829516	-10.5
26.	Z1501475009	-10.5
27.	Z1532717445	-10.5
28.	Z2037280227	-10.5
29.	Z2301684603	-10.5
30.	Z2515203810	-10.5
31.	Z27789153	-10.5
32.	Z28276117	-10.5
33.	Z3272967405	-10.5
34.	Z82272099	-10.5
35.	Z90928549	-10.5
36.	SR-1664 (control)	-10.5

**Table 2:** Physicochemical and drug likeness properties of the selected compounds.

**Compound ID**	**MW**	**ClogP**	**logS**	**HBD**	**HBA**	**TPSA**	**Rot Bonds**	**Drug likeness**	**Mut**	**Tum**	**RE**	**Irr**	**TSA**	**Molecular complexity**
Z2235802137	495.234	2.968	-4.705	3	6	97.05	14	-1.8973	X	X	X	X	371.96	0.85231
Z1982689600	456.417	3.845	-6.413	1	4	94.05	7	-10.532	X	X	X	X	335.56	0.83396
Z2235801970	472.542	1.909	-4.563	1	7	113.48	5	1.2991	X	X	X	X	362.66	0.96478
Z2037275165	410.403	2.297	-5.251	3	5	126.49	4	4.9754	X	X	X	X	286.5	0.84479
